# Proteomic discovery of prognostic protein biomarkers for persisting problems after mild traumatic brain injury

**DOI:** 10.1038/s41598-023-45965-9

**Published:** 2023-11-13

**Authors:** Min-Yong Lee, Minsoo Son, Hyun Haeng Lee, Min-Gu Kang, Seo Jung Yun, Han Gil Seo, Youngsoo Kim, Byung-Mo Oh

**Affiliations:** 1 Present Address: Department of Rehabilitation Medicine, Seoul National University Hospital, Seoul, Korea; 2 Present Address: Department of Rehabilitation Medicine, National Traffic Injury Rehabilitation Hospital, Yangpyeong, Korea; 3 Interdisciplinary Program of Bioengineering, Seoul National University College of Engineering, Seoul, Korea; 4 Mass Spectrometry Technology Access Center, McDonnell Genome Institute, Washington University School of Medicine in Saint Louis, St. Louis, MO USA; 5 Department of Rehabilitation Medicine, Konkuk University School of Medicine and Konkuk University Medical Center, Seoul, Korea; 6 Department of Rehabilitation Medicine, Seoul National University College of Medicine, Seoul, Korea; 7 Department of Biomedical Sciences, Seoul National University College of Medicine, Seoul, Korea; 8 Department of Biomedical Science, School of Medicine, CHA University, Seongnam-si, Kyeonggi-do Korea; 9 Institute on Aging, Seoul National University, Seoul, Korea

**Keywords:** Prognostic markers, Trauma, Brain injuries

## Abstract

Some individuals with mild traumatic brain injury (mTBI), also known as concussion, have neuropsychiatric and physical problems that last longer than a few months. Symptoms following mTBI are not only impacted by the kind and severity of the injury but also by the post-injury experience and the individual's responses to it, making the persistence of mTBI particularly difficult to predict. We aimed to identify prognostic blood-based protein biomarkers predicting 6-month outcomes, in light of the clinical course after the injury, in a longitudinal mTBI cohort (N = 42). Among 420 target proteins quantified by multiple-reaction monitoring-mass spectrometry assays of blood samples, 31, 43, and 15 proteins were significantly associated with the poor recovery of neuropsychological symptoms at < 72 h, 1 week, and 1 month after the injury, respectively. Sequential associations among clinical assessments (depressive symptoms and cognitive function) affecting the 6-month outcomes were evaluated. Then, candidate biomarker proteins indirectly affecting the outcome via neuropsychological symptoms were identified. Using the identified proteins, prognostic models that can predict the 6-month outcome of mTBI were developed. These protein biomarkers established in the context of the clinical course of mTBI may have potential for clinical application.

## Introduction

Traumatic brain injury (TBI) is a significant public health issue with approximately 69 million new cases worldwide each year and causing nearly 61,000 deaths in the United States^1, 2^. TBI can cause short- or long-term issues that may affect a person’s life, such as the inability to work or resume social activities^3^. Even with mild TBI (mTBI), commonly known as concussion, which accounts for 70–90% of all TBI cases, the morbidity is substantial^4^. mTBI can result in acute symptoms, including headache, dizziness, irritability, fatigue, cognitive difficulty (e.g., attention and memory), and sleep disturbances^5^. In most patients with mTBI, these symptoms resolve within days to weeks after the injury; however, some patients develop persistent physical and neuropsychiatric symptoms called post-concussion syndrome (PCS)^5^. Reportedly, up to 40% of the patients with mTBI have symptoms persisting for more than 6 months^6, 7^, and many patients with PCS experience impairments in daily life and a decrease in quality of life for an extended period, impeding their return to work or school^3, 8^.

Considering the huge personal and societal burden of mTBI, it is important to identify patients who will have sequelae of their injury and in whom early interventions would be helpful in a timely manner. Thus, a prognostic tool for outcome prediction after mTBI can aid surveillance efforts and the provision of rehabilitation services effectively and cost-efficiently^9^. Although a few models for the prediction of functional outcome after mTBI have been developed using multiple predictive factors, including demographic, socioeconomic, pre-injury and injury characteristics, imaging biomarkers, and blood biomarkers^7, 10–13^, there is still a compelling need for valid methods to predict the outcome of mTBI^7, 12^.

There has been a growing interest in the potential of biochemical biomarkers to predict mTBI prognosis^14, 15^. However, although several biomarkers, such as S100 calcium-binding protein B (S100B), glial fibrillary acidic protein (GFAP), and ubiquitin carboxy-terminal hydrolase L1 (UCH-L1), have been studied^16, 17^, no biomarker has been found that accurately predicts the outcome after mTBI. In previous studies, these biomarkers have been explored using a hypothesis-driven approach^18^, with limited success. Therefore, data-driven approaches such as microRNA arrays or proteomics that can complement this traditional approach have been suggested^18^. Particularly, mass spectrometry-based proteomics can precisely quantify hundreds of proteins from a complex mixture of samples, enabling the identification of more useful biomarkers^19^. However, a limitation of mTBI biomarker research is that the majority of studies have reported values of biomarkers taken at a single time point after injury^15^. In mTBI, studying changes in biomarkers over a short time window is suboptimal because patients present to a clinic or hospital at various stages after injury^20^. Thus, it is important to perform longitudinal studies over time after mTBI^15^. A longitudinal study could provide a useful background to identify candidate prognostic biomarkers of mTBI.

Therefore, we aimed to identify candidate prognostic biomarkers of recovery from mTBI using mass spectrometry-based proteomics and develop a predictive model for prognosis at 6 months after injury in a prospective longitudinal cohort of patients with mTBI.

## Results

### Patient characteristics

A total of 48 patients were enrolled during the 6-month follow-up, and 42 patients were analyzed in the study (six patients were excluded because of a missed follow-up beyond 1 week after injury [n = 2] or failure to collect serum samples [n = 4]). Most of the 42 participants were previously healthy, except for 6 participants with a history of hypertension or diabetes. Neuropsychological assessments, such as the Beck Depression Inventory-II (BDI-II), Korean-Montreal Cognitive Assessment (K-MoCA), and Frontal Assessment Battery (FAB) scores, and the serum samples at four time points after injury (< 72 h, 1 week, 1 month, and 3 months) from the 42 patients were used to identify the candidate prognostic biomarkers for recovery from mTBI.

For prognostic classification, the patients were classified into the poor recovery (PR) or good recovery (GR) groups based on the outcomes (assessed using the Rivermead Post-concussion Symptoms Questionnaire [RPCQ] and Glasgow Outcome Scale-Extended [GOSE] at 6 months). The RPCQ and GOSE measure the subjective distress associated with diverse physical, emotional, and cognitive symptoms and the level of daily function, respectively. Overall, three of the 42 patients who had follow-up information only for 3 months (n = 1) or were not followed up at 3 or 6 months (n = 2) were excluded from the prognostic classification. The serum samples from 22 patients in the GR group and 17 patients in the PR group were used for developing a prognostic model (Table 1). The PR and GR prognostic groups differed significantly with regard to RPCQ scores at 1 week (Student’s t-test, *P* = 0.0016, respectively), GOSE scores at 3 months (Student’s t-test, *P* = 0.0026, respectively), and BDI-II scores at 3 months and 6 months (Student’s t-test, *P* = 0.0022 and *P* = 2.28e−5, respectively). The 1- and 3-month K-MoCA and FAB scores were not significantly different between the PR and GR groups.

**Table 1:** Summary of the clinical information of the study cohort.

Characteristics	All^a^ (N = 42)	For regression analysis (N = 39)		
		Good recovery (N = 22)	Poor recovery (N = 17)	*P* value
Age at inclusion, years, Mean (SE)	50.6 (2.8)	47.0 (3.7)	52.0 (4.3)	0.38
Sex, count (%)				
Female	19 (45.2)	9 (40.9)	9 (52.9)	0.67
Male	23 (54.8)	13 (59.1)	8 (47.1)	
Time from injury to enrollment (days), mean (SE)	9.6 (2.4)	6.2 (1.4)	11.0 (4.1)	0.23
Mechanism of injury, Count (%)				
Fall	31 (73.8)	17 (77.3)	12 (70.6)	0.70
Traffic accident	8 (19.0)	3 (13.6)	4 (23.5)	
Others	3 (7.1)	2 (9.1)	1 (5.9)	
CT abnormality^b^, Count (%)				
Yes	7 (16.7)	2 (9.1)	3 (17.6)	0.77
No	35 (83.3)	20 (90.9)	14 (82.4)	
LOC, Count (%)				
Yes	32 (76.2)	16 (72.7)	14 (82.4)	0.75
No	10 (23.8)	6 (27.3)	3 (17.6)	
PTA, Count (%)				
Yes	19 (45.2)	9 (40.9)	8 (47.1)	0.94
No	22 (52.4)	12 (54.6)	9 (52.9)	
Unknown	1 (2.4)	1 (4.5)	0 (0.0)	
RPCQ, Mean (SE; N)				
At 1 week	16.8 (2.1; 31)	12.2 (2.3; 17)	25.3 (3.0; 12)	0.0016
At 1 month	19.5 (2.6; 40)	14.0 (2.7; 22)	29.4 (4.0; 16)	0.0022
At 3 months	18.5 (2.3; 40)	11.3 (2.1; 22)	26.8 (3.5; 17)	< 0.001
At 6 months	14.7 (2.3; 34)	5.6 (1.1; 19)	26.3 (3.1; 15)	< 0.001
GOSE, Mean (SE; N)				
At 1 month	6.4 (0.2; 40)	6.6 (0.2; 22)	5.9 (0.2; 16)	0.062
At 3 months	6.8 (0.1; 40)	7.2 (0.2; 22)	6.3 (0.2; 17)	0.0026
At 6 months	6.8 (0.2; 34)	7.5 (0.2; 19)	6.9 (0.3; 15)	< 0.001
BDI-II, Mean (SE; N)				
At 1 month	12.1 (1.7; 40)	10.6 (2.6; 22)	15.1 (2.0; 16)	0.20
At 3 months	11.7 (1.5; 40)	7.5 (1.6; 22)	15.9 (2.0; 17)	0.0022
At 6 months	7.4 (1.3; 34)	2.5 (0.7; 19)	13.5 (1.8; 15)	< 0.001
K-MoCA, Mean (SE; N)				
At 1 month	24.3 (0.7; 40)	25.0 (1.0; 22)	23.4 (1.1; 16)	0.32
At 3 months	25 (0.6; 40)	25.5 (0.8; 22)	24.4 (1.0; 17)	0.39
FAB, Mean (SE; N)				
At 1 month	15.7 (0.4; 40)	15.5 (0.7; 22)	16.1 (0.3; 16)	0.47
At 3 months	16.5 (0.4; 40)	16.8 (0.4; 22)	16.1 (0.7; 17)	0.39

SE, standard error; CT, computed tomography; LOC, loss of consciousness; PTA, post-traumatic amnesia; RPCQ, Rivermead Post-concussion Symptoms Questionnaire; GOSE, Glasgow Outcome Scale-Extended; BDI-II, Beck Depression Inventory-II; K-MoCA, Korean- Montreal Cognitive Assessment; FAB, Frontal Assessment Battery.

^a^Three of 42 patients were excluded from the recovery group because of follow-up only at 3 months (1 case) or no follow-up between 3 and 6 months. The three cases were only included in serial mediation and moderation analysis.

^b^Specific information regarding abnormal computed tomography findings are described in Supplementary Results.

### Association of longitudinal proteomic alterations in the serum with the 6-month outcomes

To select analytic targets, we compiled a total of 3130 candidates of target proteins from three comprehensive TBI animal model studies using RNAseq techniques with well-known TBI biomarker lists (Supplementary Table S1). Among them, we selected 763 peptides representing 528 proteins as detectable targets in plasma. Out of 528 proteins, 420 proteins were used for data processing, according to interference-free and skewness in overall samples (Supplementary Methods and Supplementary Table S2).

**Figure 1:**
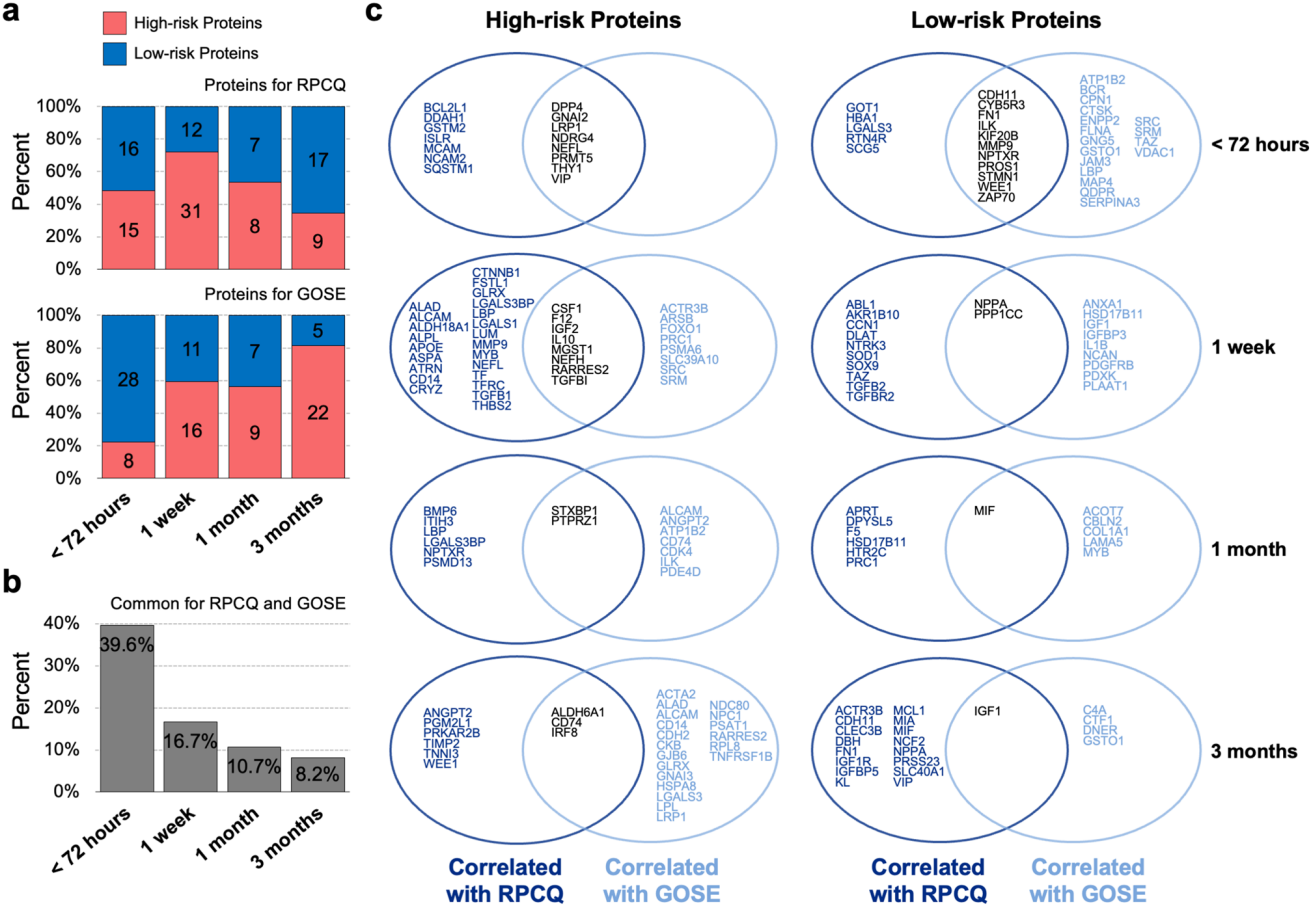
Linear association of protein levels at each phase to prognostic outcomes. (**a**) The proportion of high- and low-risk proteins significantly associated with RPCQ and GOSE outcomes, within 72 h, and at 1 week, 1 month, and 3 months. The labels on the bar represent the counts of the proteins. (**b**) The percentage of common proteins between high- and low-risk proteins at each time point. (**c**) A Venn diagram of high- and low-risk proteins associated with for RPCQ and GOSE score at each time point. Gene name of proteins were marked. RPCQ, Rivermead Post-concussion Symptoms Questionnaire; GOSE, Glasgow Outcome Scale-Extended.

To select a candidate prognostic biomarker of mTBI, linear regression analysis was performed to verify the association between 6-month outcomes (RPCQ and GOSE scores) and plasma levels of 420 qualified proteins at the four time points. Proteins associated with a higher risk for poor prognosis (positive correlation with the RPCQ scores or negative correlation with the GOSE score) were classified as high-risk proteins. In contrast, proteins associated with a lower risk of poor prognosis were classified as low-risk proteins. In the regression analysis of the RPCQ score at 6 months, 31 proteins (high-risk protein/low-risk protein: 15/16) within 72 h, 43 proteins (31/12) at 1 week, 15 proteins (8/7) at 1 month, and 26 proteins (9/17) at 3 months showed a significant association. Additionally, 36 proteins (high-risk protein/low-risk protein: 8/28) within 72 h, 27 proteins (16/11) at 1 week, 16 proteins (9/7) at 1 month, and 27 proteins (22/5) at 3 months were significantly associated with the GOSE score at 6 months (Fig. 1a).

The characteristics of the selected proteins are summarized as follows: (1) within 72 h, low-risk proteins were more enriched than high-risk proteins by 3.5-fold relative to the GOSE score; (2) at 1 week, high-risk proteins were more enriched than low-risk proteins, relative to both RPCQ (2.6-fold) and GOSE scores (1.5-fold); (3) at 3 months, low-risk proteins for the RPCQ score were more enriched than high-risk proteins by 1.9-fold, whereas high-risk proteins associated with the GOSE score were more enriched than low-risk proteins by 4.4-fold; and (4) the percentage of common proteins associated with the RPCQ and GOSE scores decreased sequentially over time, implying that more different biological events affected the RPCQ and GOSE scores at later time points (Fig. 1b). The list of proteins associated with changes in the RPCQ and GOSE scores is presented in Fig. 1c.

### Serial mediation between the neuropsychological assessment and the 6-month outcomes

To verify the clinical factors affecting the prognosis of mTBI, linear regression analysis was performed between clinical assessments before 6 months and outcomes (RPCQ and GOSE scores) at 6 months (Supplementary Table S3). The RPCQ score at 6 months was significantly associated with the K-MoCA score at 3 months (F-test, *P* = 0.029), and the GOSE score at 6 months was significantly associated with the K-MoCA scores at 1 month (F-test, *P* = 0.013) and 3 months (F-test, *P* = 0.0088) and FAB scores at 1 month (F-test, *P* = 0.027) and 3 months (F-test, *P* = 2.8e−4).

**Figure 2:**
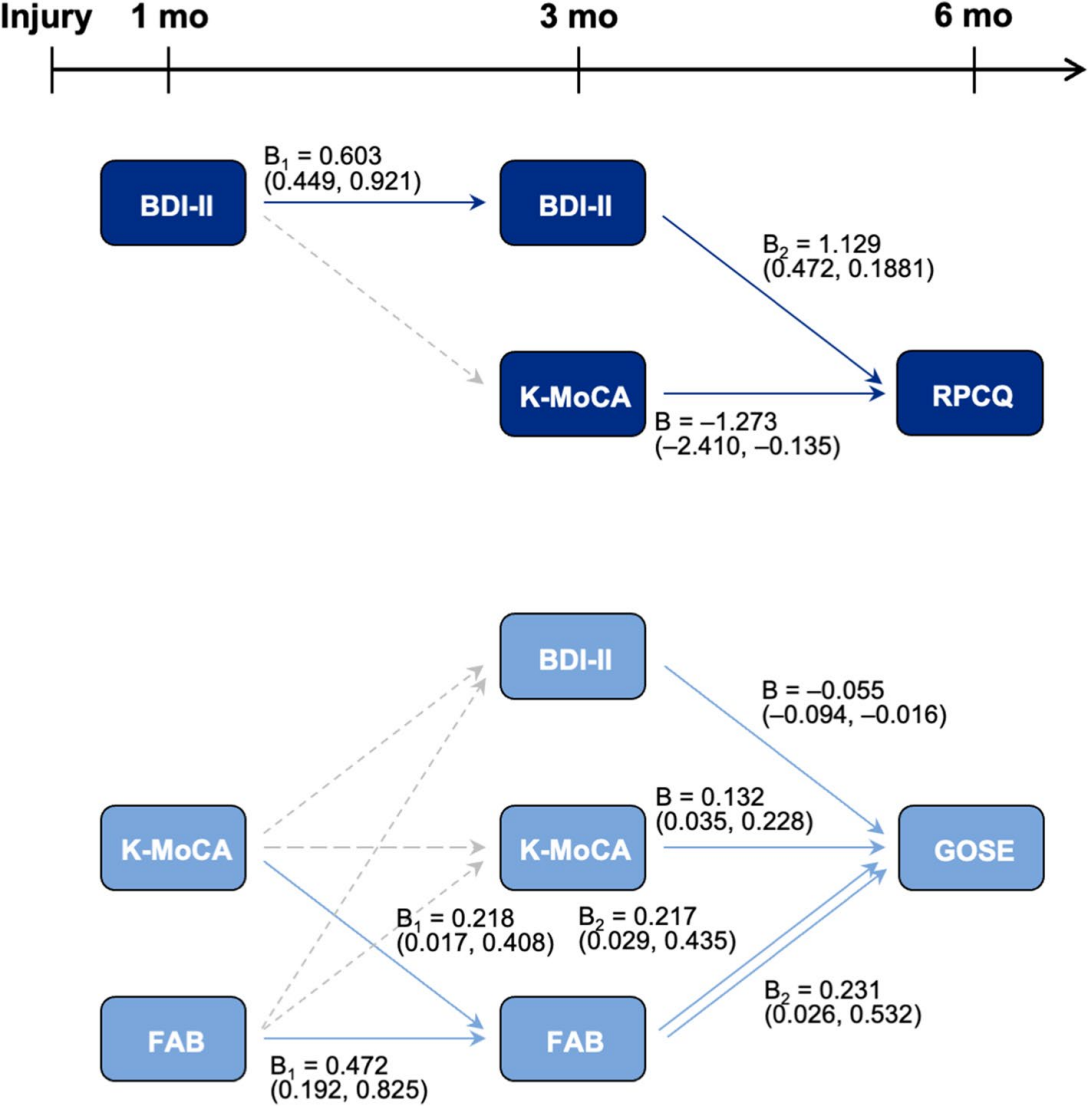
Serial mediation between neuropsychological assessments over time after mild traumatic brain injury. Indirect or direct effects of neuropsychological assessments on post-concussive symptoms (RPCQ) and functional level (GOSE) at 6 months. The neuropsychological assessment findings, which were significantly correlated with outcomes (RPCQ and GOSE scores) in the linear regression analysis, are depicted. The results of the mediation analysis are marked with their estimate and 95% confidence interval, but the results of linear regression analysis are marked if a significant indirect or direct effect was found. The solid arrows indicate valid indirect or direct effects. The dotted arrows indicate that a significant effect was not found between variables. BDI-II, Beck Depression Inventory-II; K-MoCA, Korean-Montreal Cognitive Assessment; RPCQ, Rivermead Post-concussion Symptoms Questionnaire; FAB, Frontal Assessment Battery; GOSE, Glasgow Outcome Scale-Extended.

To investigate the causal chain linking the mediators between clinical symptoms and time progression that affected outcomes at 6 months, a serial mediation analysis was performed. The RPCQ score at 6 months was indirectly affected by the BDI-II scores at 1 month and 3 months (B = 0.680, bootstrapped 95% confidence interval [CI], 0.230–1.494) and only directly influenced by the K-MoCA score at 3 months. The GOSE score at 6 months was indirectly affected by the K-MoCA and FAB scores at 1 month and FAB score at 3 months (K-MoCA: B = 0.047, bootstrapped 95% CI, 0.001–0.114; FAB: B = 0.109, bootstrapped 95% CI, 0.009–0.313). The BDI-II and K-MoCA scores at 3 months influenced the GOSE score at 6 months only directly (Fig. 2).

### Prognostic indirect effects of biological events

**Figure 3:**
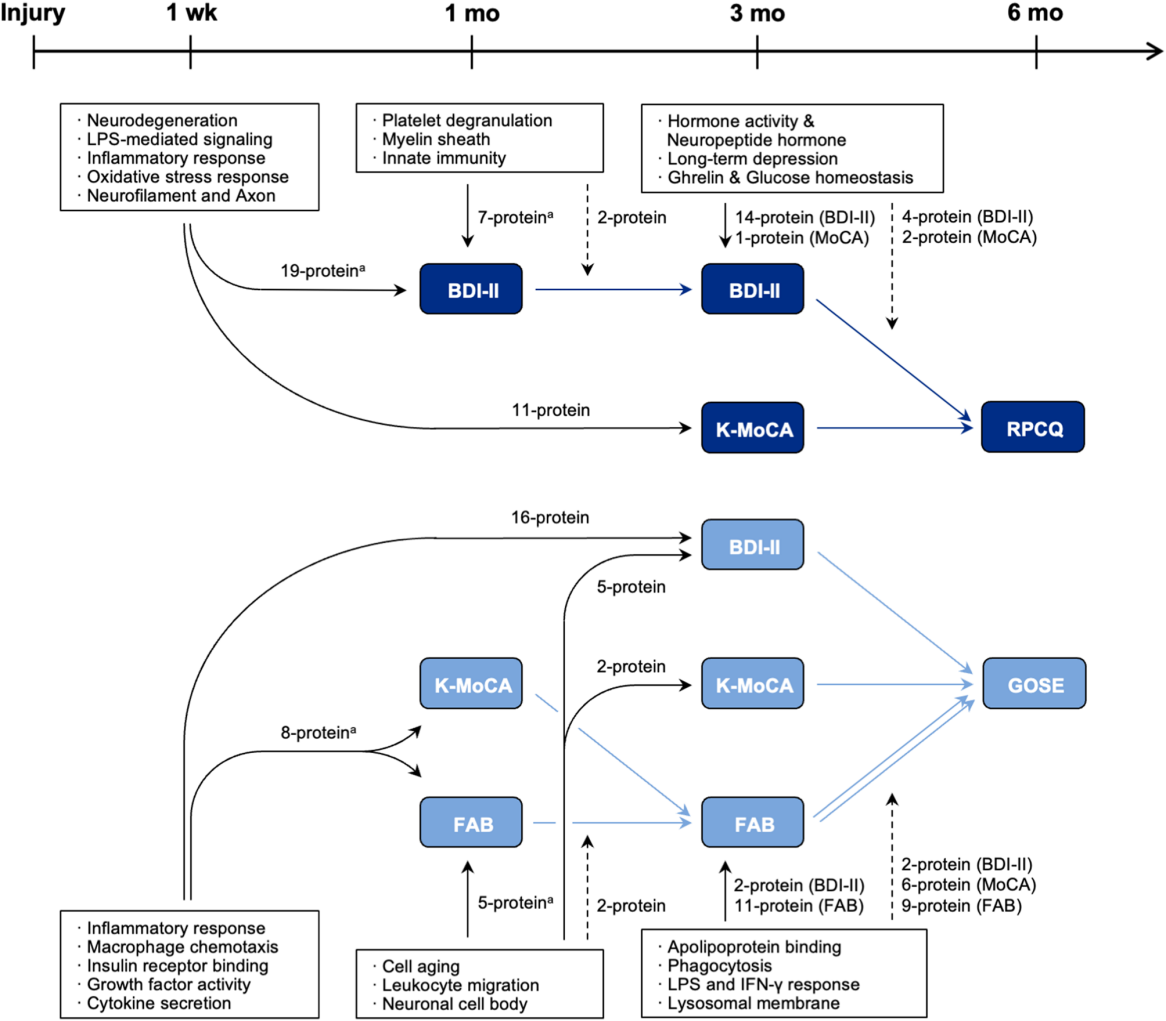
Prognostic indirect effects of biological events in the chronic phases after mild traumatic brain injury. Indirect effects of protein levels at 1 week, 1 month, and 3 months to the serial mediation between neuropsychological assessments in Fig. 2. Only neuropsychological assessment findings that showed significant association with prognostic outcomes are depicted. The solid arrows represent serial mediation between variables, and the dotted arrows represent moderated mediation. The representative biological terms, enriched by the proteins indirectly affecting prognostic outcomes, are marked in the boxes. All significant indirect effects of protein levels are depicted, except for the indirect effect of “protein level in 1 week – K-MoCA – GOSE” by 2-protein due to its lower importance. BDI-II, Beck Depression Inventory-II; K-MoCA, Korean-Montreal Cognitive Assessment; RPCQ, Rivermead Post-concussion Symptoms Questionnaire; FAB, Frontal Assessment Battery; GOSE, Glasgow Outcome Scale-Extended. ^a^To test the indirect effect of proteins via dual mediators, each mediator was tested separately.

To investigate which biological events affected the prognostic outcome at 6 months via or with neuropsychological symptoms (BDI-II, K-MoCA, and FAB scores), serial mediation and moderation analyses were performed using the proteins associated with the RPCQ or GOSE scores at 6 months in the linear regression analysis (Fig. 3 and Supplementary Table S4).

Among early (at 1 week) proteins that were associated with prognostic outcomes, 19 proteins indirectly affected the RPCQ score at 6 months through the BDI-II path and 11 proteins via the K-MoCA path. The GOSE score at 6 months was affected by proteins at 1 week via the BDI-II (16 proteins), K-MoCA (2 proteins), and FAB (8 proteins) paths (Fig. 3). The biological terms from the gene ontology functional annotation analysis were enriched by the proteins mediating each path as follows (Supplementary Fig. 1a and b and Supplementary Table S5): (1) in the BDI-II path, inflammatory terms, such as inflammatory response, macrophage chemotaxis, growth factor activity, and cytokine secretion, and terms related to apoptosis, such as mitochondrion, reactive oxygen species metabolic process, and regulation of apoptotic process, were enriched for both RPCQ and GOSE scores; (2) the terms related to axon and neurofilament were enriched in the K-MoCA path for the RPCQ score; (3) any meaningful term was not enriched in the K-MoCA path for the GOSE score; and (4) in the FAB path for the GOSE score, inflammatory and apoptosis terms were enriched, similar to the results of the BDI-II path.

At 1 month and 3 months after injury, the proteins that indirectly affected prognosis or moderated mediation of neuropsychological paths to the prognostic outcome were selected very differently between the RPCQ and GOSE scores (Supplementary Fig. 1c–e, and Supplementary Table S5). The characteristics of the selected proteins are summarized as follows: (1) 8 proteins at 1 month were associated with the RPCQ score only through the BDI-II path, and the biological terms related to myelin sheath and innated immunity were enriched; (2) the proteins at 1 month were associated with the GOSE score through all the BDI-II (5 proteins), K-MoCA (2 proteins), and FAB (5 proteins) paths, but the biological terms associated with mTBI, such as cell aging and neuronal cell body, were enriched only in the FAB paths; (3) at 3 months, proteins were associated with the RPCQ score mainly via the BDI-II path, and the terms related to hormones, depression, Ghrelin, and glucose homeostasis were enriched; and (4) for the GOSE score, the FAB path was the main route of the proteins related to lipoprotein, phagocytosis, and lipopolysaccharide interferon-γ (LPS-IFNγ) (Fig. 3).

Considering the reduction of common risk proteins between the RPCQ and GOSE scores seen in Fig. 1, we hypothesized that some events in the acute phase would influence both RPCQ and GOSE scores simultaneously, but the two outcomes occur via different paths of the events in later phases. Thus, 19 of 48 risk proteins associated with both prognostic outcomes were selected to demonstrate the association of acute events with prognosis. To verify whether the selected proteins were associated with mTBI, a functional association network between 19 proteins and the two well-known mTBI biomarkers, GFAP and UCH-L1, was examined, and significant protein–protein interactions were confirmed between the proteins, except for CYB5R3 (Supplementary Fig. 2a; *P* = 3.48e−7). Moreover, the acute protein factor, which was the first component calculated from factor analysis using 18 proteins, excluding CYB5R3, showed a significant correlation with the RPCQ score at 6 months (linear regression analysis: R^2^ = 0.81 and *P* = 1.18e−5) and GOSE score at 6 months (linear regression analysis: R^2^ = 0.90, *P* = 2.81e−7) (Supplementary Fig. 2b). Through the functional annotation analysis using the chronic phase proteins correlated with the acute protein factor, we could verify the effect of acute events on the biological terms enriched at 1 week, 1 month, and 3 months (Supplementary Fig. 2c–e, Supplementary Table S6, and Supplementary Methods).

### Predicting outcomes using protein biomarker panels

To assess the prognosis of patients with mTBI (GR vs. PR) using plasma protein levels in an objective manner, logistic regression analysis was performed at each time point (39 patients). Univariate analysis of the proteins indirectly affecting prognostic outcomes showed an association with PR (Wald test, *P* < 0.1), with 12 of 18 proteins (66.7%) within 72 h, 12 of 16 proteins (75.0%) at 1 week, all 4 proteins (100.0%) at 1 month, and 6 of 13 proteins (46.2%) at 3 months (Supplementary Table S7). Multivariate logistic regression analysis with backward selection by 10 iterations of threefold cross-validation showed that microsomal glutathione S-transferase 1, neurofilament heavy chain, and retinoic acid receptor responder 2 levels at 1 week; activated leukocyte cell adhesion molecule, macrophage migration inhibitory factor, and syntaxin-binding protein 1 at 1 month; and insulin-like growth factor-binding protein 5 (IGFBP5), interferon regulatory factor 8, and lipoprotein lipase at 3 months were independent prognostic factors at 6 months (Supplementary Table S7). We could not find independent variables within 72 h because of the small sample size (N = 14). This prognostic stratification scheme classified the PR group with approximately 80.0% accuracy and area under the curve and could assess the biological events associated with prognosis (Table 2 and Supplementary Fig. 3). The longitudinal expression of protein variables in the models is shown in Supplementary Fig. 4.

**Table 2:** Predictive performance of the regression models at the four time points.

Time point	Patient, n	Protein name	Association with outcome	Associated biological terms	Accuracy (95% CI)	AUC (95% CI)
< 72 h	14	DPP4^a^	Both	Acute events	0.80 (0.74–0.86)	0.83 (0.75–0.90)
		LRP1^a^	Both	Acute events	0.84 (0.78–0.90)	0.79 (0.70–0.87)
		THY1^a^	Both	Acute events	0.85 (0.80–0.90)	0.82 (0.74–0.89)
1 week	29	MGST1	Both	Mitochondrion	0.83 (0.79–0.90)	0.81 (0.76–0.87)
		NEFH	Both	Axon, neurofilament, mitochondrion		
		RARRES2	Both	Inflammation		
1 month	38	ALCAM	GOSE score	Neuronal cell body,	0.82 (0.79–0.85)	0.83 (0.79–0.88)
		MIF	Both	myelin sheath, cell aging		
		STXBP1	RPCQ score	Myelin sheath		
3 months	36	IGFBP5	RPCQ score	Glucose, homeostasis	0.86 (0.82–0.89)	0.90 (0.86–0.93)
		IRF8	GOSE score	LPS-IFNγ		
		LPL	GOSE score	Lipoprotein		

CI, confidence interval; AUC, area under the curve; RPCQ, Rivermead Post-concussion Symptoms Questionnaire; GOSE, Glasgow Outcome Scale-Extended; Both, GOSE and RPCQ scores; MGST1, microsomal glutathione S-transferase 1; RARRES2, retinoic acid receptor responder 2; ALCAM, activated leukocyte cell adhesion molecule; MIF, macrophage migration inhibitory factor; STXBP1, syntaxin-binding protein 1; IGFBP5, insulin-like growth factor-binding protein 5; IRF8, interferon regulatory factor 8; LPL, lipoprotein lipase; NEFH, neurofilament heavy chain; THY1, Thy-1 cell surface antigen; LRP1, low-density lipoprotein receptor-related protein 1; DPP4, dipeptidyl peptidase-4.

^a^Univariates in logistic regression analysis.

## Discussion

In this prospective study, we identified candidate prognostic biomarkers using proteomics and developed prognostic models at 6 months after injury in a longitudinal cohort of 42 patients with mTBI. First, proteins significantly associated with the 6-month outcome were selected among the proteins quantified by the highly quantitative multiple reaction monitoring-mass spectrometry (MRM-MS) assays (Fig. 1). Next, we identified sequential associations among clinical assessments affecting the 6-month outcome of mTBI (Fig. 2). Then, we identified candidate prognostic biomarker proteins that indirectly affected the 6-month outcome via neuropsychological symptoms (BDI-II or K-MoCA and FAB paths) (Fig. 3). Using the candidate biomarker proteins, the prognostic models for each phase that can predict the 6-month outcome of mTBI were developed (Table 2).

**Figure 4:**
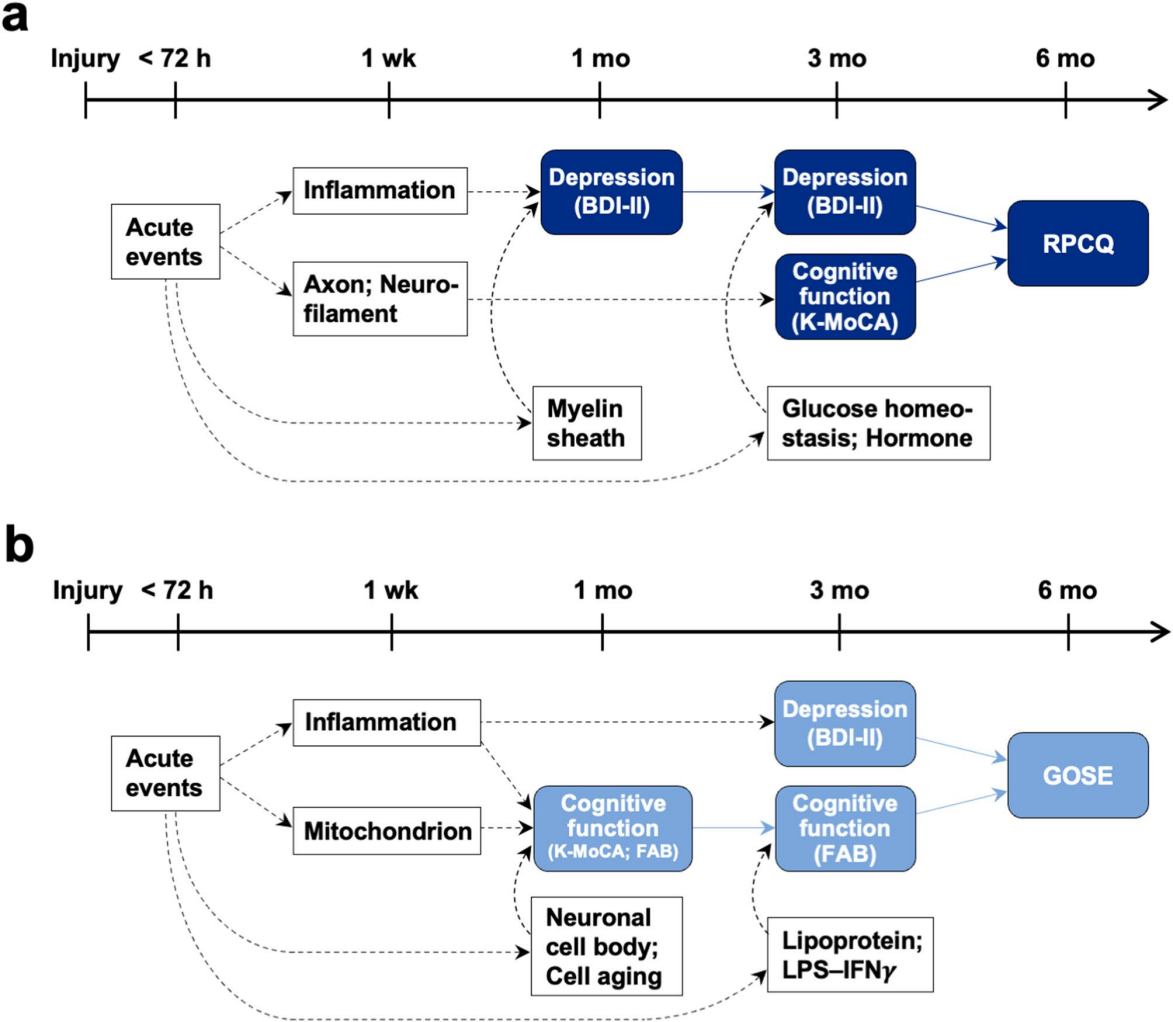
Sequential associations between biological events and neuropsychological assessments after mild traumatic brain injury. Summary of longitudinal effects of biological events on the RPCQ (**a**) and GOSE scores (**b**) via neuropsychological symptoms after head trauma followed by acute events. Clinical assessments are depicted in the blue boxes, and biological events are shown in the white boxes. Only biological terms that showed a correlation with the acute protein factor are displayed. The solid arrows represent the effects between the clinical assessment findings, and the dotted arrows indicate the effects from biological events. The longitudinal timeline of follow-up is depicted on the Y-axis with ticks at each time point. BDI-II, Beck Depression Inventory-II; K-MoCA, Korean-Montreal Cognitive Assessment; RPCQ, Rivermead Post-concussion Symptoms Questionnaire; FAB, Frontal Assessment Battery; GOSE, Glasgow Outcome Scale-Extended.

By analyzing the biological terms of these prognostic candidate biomarker proteins, we could determine implications for the pathophysiology of mTBI. The sequence from the events that occurred within 72 h after injury to the prognostic outcomes at 6 months is summarized as follows (Fig. 4): (1) acute events caused inflammation during the first week, followed by depressive symptoms, which worsened the RPCQ and GOSE scores at 6 months; (2) at 1 week, the secretion of proteins from axons and neurofilament to plasma was associated with a poor RPCQ score via impaired cognitive function tested by the K-MoCA, whereas the mitochondrial proteins related to inflammation were associated with a poor GOSE score via cognitive decline tested by the FAB; (3) the effect of depression on the RPCQ score varied depending on the myelin sheath degradation at 1 month and alterations in the hormone levels and glucose homeostasis at 3 months; and (4) the effect of cognitive decline (FAB score) on the GOSE score varied depending on neuronal damage at 1 month and alterations in lipoproteins and LPS-IFNγ proteins at 3 months.

At 1 week, the main biological term associated with the outcome was inflammation. Inflammation has an important role in the pathophysiology of secondary brain injury after TBI^21^. Since post-traumatic inflammatory response plays an important role in neurodegeneration, corticosteroids were used after TBI until the early 21th century, when the CRASH trial was published^22^. Although the CRASH trial showed that corticosteroids should not be used routinely after TBI, this does not negate the role of inflammation in TBI. The neuroinflammation following mTBI has been hypothesized to be associated with post-concussive symptoms^23, 24^. An interesting study reported that initially elevated C-reactive protein levels might be an independent predictor of persistent PCS in patients with mTBI^25^. Similar results that inflammation may influence the outcome, mediating depressive symptoms and cognitive function, were found in this study. However, further investigation of the role of inflammation in the sequelae following mTBI is required.

Axon and neurofilament proteins in the plasma at 1 week showed a correlation with RPCQ at 6 months via cognitive decline at 3 months in this study. The subsequent axonal damage is one of the most common pathological mechanisms of long-term dysfunction in mTBI^21^. A few studies have reported that cognitive dysfunction can correlate with the extent of microstructural white matter damage^21, 23^. Thus, axon-related proteins, such as neurofilament light (NFL), spectrin N-terminal fragment (SNTF), and A-tau, have been extensively studied as biomarkers of TBI^26^. In this study, candidate biomarkers discovered at 1 week were neurofilament heavy and light polypeptides, which is consistent with other studies. Activated leukocyte cell adhesion molecule (ALCAM) was also discovered at 1 week. ALCAM is known to play a role in cell adhesion, axon growth, axonal pathfinding, and neuronal migration and differentiation^27^. There is a paucity of studies on ALCAM as a TBI biomarker; however, it may have potential as a candidate biomarker in TBI. Mitochondrion was also identified in the subacute phase (1 week) as a biological term that can influence the outcome of mTBI via cognitive decline. Mitochondrial dysfunction has been reported in animal models and humans and is considered to be an acute response after TBI^28, 29^. The primary function of mitochondria is the production of adenosine triphosphate (ATP) and reactive oxygen species^30^. Thus, mitochondria dysfunction can exacerbate the cellular energy crisis and aggravate cell damage^28, 31^. This energy crisis can impact post-concussive symptoms and increase the vulnerability to second impact syndrome in mTBI patients^31, 32^.

At 1 month, the effect of depression on the 6-month RPCQ score varied depending on the degradation of the myelin sheath. Myelin surrounds and protects axons in the central nervous system (CNS). After TBI, myelin damage can result from axon injury, neuronal cell death, or secondary damage that causes oligodendrocyte loss with subsequent demyelination of intact axons^33^. Although the role of myelin injury in the pathophysiology of mTBI remains poorly understood, it is known that myelin damage contributes to white matter injury along with axonal injury^34, 35^; thus, myelin can be associated with cognitive function^36^. However, the present study differs in that myelin showed an association with depressive symptoms. Although the association between depressive symptoms and decreased white matter integrity in multiple brain areas after mTBI was reported in a previous study^37^, it needs further investigation.

Moreover, microglial activation terms, such as neuronal damage, were associated with 6-month GOSE via cognitive decline at 1 month. Microglia are the immune cells of the CNS that have roles in brain inflammation through their interaction with white matter^38, 39^. Microglial activation has recently been of increasing interest because they have been recognized as a key regulator of degeneration and regeneration of the white matter^39^. As white matter injury is known to be a major contributor to the impairments associated with mTBI, microglial activation has been studied as a biomarker and a potential target of therapeutics in mTBI^39, 40^. Although the results of the present study might be supportive of this, there is still much to be revealed.

At 3 months, alterations in lipoproteins and LPS-IFNγ were associated with the 6-month outcome via cognitive decline. Among lipoproteins, lipoprotein receptor protein 1 (LRP1) was a candidate biomarker identified in this study. LRP1, a regulator of blood–brain barrier (BBB) integrity, may have roles in dementia progression^41, 42^. TBI-induced BBB dysfunction has been reported in previous studies^42^. However, there is a paucity of studies examining the role of LRP1 in TBI. Meanwhile, IFN-γ may play a significant role in the pathophysiology of TBI as a neuroinflammatory mediator^43^. A study reported that IFN-γ level in mTBI patient group was significantly increased compared with that in the community control group at 1 year after injury, suggesting an activation of the innate immune system up to 1 year post-injury^44^. Although our study also showed similar results, further research is needed.

In addition, glucose homeostasis showed a correlation with RPCQ at 6 months, mediating depressive symptoms at 3 months. In this study, the candidate biomarker protein related to glucose homeostasis was IGFBP5, which can exert both stimulatory and inhibitory effects on IGF-1 signaling^45^. Although IGF-1 exhibits both neuroprotective and neuroregenerative effects after TBI^46^, further studies are needed on the potential of IGFBP5 as a mTBI biomarker.

Because of the heterogeneity of mTBI, it is difficult to predict the prognosis of the patients. Until recently, several studies have attempted to predict the prognosis using multiple factors^7, 47, 48^. These models were mainly based on clinical factors, such as admission characteristics, post-injury symptoms, and psychological symptoms. However, in an external validation study using the Collaborative European NeuroTrauma Effectiveness Research in Traumatic Brain Injury (CENTER-TBI) data, none of these predictive models showed good calibration and discrimination capabilities in patients with mTBI^7^.

Blood-based biomarkers have been proposed as novel predictors of mTBI prognosis. Commonly studied mTBI prognostic biomarkers, such as GFAP, S100B, neuron-specific enolase, and UCH-L1, have shown poor discriminating ability^18^. Ghrelin, NFL, SNTF, and A-tau have been studied as potential biomarkers for the prognosis of mTBI^26, 49–51^. For example, a recent study that was conducted to predict recovery after sports-related concussion in professional flat-track jockeys using blood biomarkers, such as GFAP, NFL, and tau, suggested that cognitive testing and blood biomarkers may be potential objective measures to assist in the monitoring of concussion recovery^52^. However, the relation of ghrelin, NFL, SNTF, and A-tau is limited to axonal injury, which is one of the most common pathological mechanisms of long-term dysfunction in mTBI^21^. The pathophysiology of mTBI is highly complex; therefore, it is necessary to discover various candidate biomarkers that can comprehensively cover the pathophysiology.

Unlike previous studies of mTBI biomarkers that used a hypothesis-driven approach, we utilized proteomics, a data-driven approach, to discover novel prognostic candidate biomarkers. Although the hypothesis-driven approach is less likely to yield false-positive results, it is limited by current understanding and the time it requires^18^. In data-driven methods such as proteomics, a large number of candidate biomarkers can be discovered quickly because candidate biomarkers can be screened without regard to the pathophysiology, although it is more likely to yield false-positive results^53^. To reduce false-positive results of proteomics, serial mediation and moderation analyses were performed in this study. Although cross-sectional approaches to mediation are possible to generate biased estimates of longitudinal parameters in the special case of complete mediation^54^, serial mediation analysis is powerful and widely used to find mediators between variables. We investigated a causal chain linking the mediators between clinical symptoms and time progression and then selected the proteins that mediated these causal linking chains and influenced the 6-month outcome. Thus, the selected proteins might be more reliable as mTBI prognostic biomarkers.

This study identified candidate mTBI prognostic biomarkers at multiple time points from the early to the chronic phase after injury, whereas previous studies have mostly identified biomarkers only at a single time point. A biomarker that is available in the acute phase of injury is not appropriate in the chronic phase. For example, the level of GFAP in peripheral blood reaches a peak at 20 h after trauma but is detected at a very low concentration after 72 h^17^. Biomarkers such as GFAP can be used for diagnosis or prediction of the outcome in the acute phase of injury, but it is difficult to use them after 3 days of injury. Because patients with mTBI may not visit the hospital in the acute stage of the injury alone, the candidate prognostic biomarkers at various time points identified in this study are expected to be clinically useful.

The present study used both the RPCQ and GOSE as outcomes for developing prognostic models of mTBI. Although there is an overlap between both measures, there is also a difference in that RPCQ reflects patients’ discomfort, while GOSE reflects the overall function. Therefore, in previous studies, post-concussive symptoms measured by RPCQ or GOSE at 6 months after injury have been used as the outcome of mTBI^7^. However, both measures have limitations as outcome variables. Although RPCQ is employed most often in measuring post-concussive symptoms^55^, there is no guidance for assessing PCS using RPCQ. GOSE is widely employed as a primary outcome measure in TBI studies, but its utility as an outcome measure in patients with mTBI is controversial because GOSE is not sensitive enough to find different health problems despite good functioning^7^. Therefore, to complement these limitations, we used both outcomes of mTBI for developing prognostic models in this study.

This study has some limitations. This study only includes a single time point (3 months) in the early chronic phase. It would be better to conduct blood sample collection and clinical assessments between 1 and 3 months and beyond the 3-month period. The validity of the serial use of assessment tools has not yet been validated. Measurements at some time points were missed in a few participants because of the time of their enrollment. In particular, we could not find the independent variables in the logistic regression due to a lower number of blood samples within 72 h after trauma and could find only single variables. To enhance patient care in the emergency department (ED), biomarkers that can be applied within 72 h should be identified in further studies. For dichotomization of the RPCQ at 6 months, we matched 16 post-concussive symptoms of the RPCQ to 8 symptoms of the ICD-10 criteria. Although it is not suitable for diagnosis of PCS because the RPCQ is a questionnaire, several studies have employed ICD-10 matching^47, 56^. Another limitation is that the use of mass spectrometry data requires sophisticated software and expertise, and data interpretation can be complex and prone to errors. In addition, there may be a selection bias toward more severe patients because this study was conducted at a tertiary hospital. A future multicenter study would ensure the representativeness of the sample group and a larger sample size.

In conclusion, we identified prognostic candidate biomarkers using proteomics in light of clinical course after mTBI. Although our results should be externally validated for generalizability, the established protein biomarkers in the clinical course of mTBI may have potential for clinical application.

## Methods

### Ethics statements

The ethics committee of Seoul National University Hospital, Korea, approved the study (1603-147-751 and 1805-079-947). Written informed consent was obtained from all participants. All methods were carried out in accordance with relevant guidelines and regulations.

### Study design and population

This prospective longitudinal cohort study enrolled patients with head trauma at the ED or outpatient clinic of Seoul National University Hospital from January 2018 to January 2021. The inclusion criteria were as follows: (1) age ≥ 18 years, (2) patients presenting within 1 month after head trauma, and (3) diagnosis of mTBI. The diagnosis of mTBI was based on the World Health Organization diagnostic criteria:

“Operational mTBI criteria for clinical identification include (a) 1 or more of the following: confusion or disorientation, loss of consciousness for 30 min or less, post-traumatic amnesia for less than 24 h, and/or other transient neurological abnormalities such as focal signs, seizure, and intracranial lesion not requiring surgery; (b) Glasgow Coma Scale score of 13–15 after 30 min post-injury or later upon presentation for health care. These manifestations of mTBI must not be caused by drugs, alcohol, caused by other injuries or treatment for other injuries (e.g., systemic injuries, facial injuries, or intubation), caused by other problems (e.g., psychological trauma, language barrier, or coexisting medical conditions), or caused by penetrating craniocerebral injury”^57^.

The exclusion criteria were as follows: (1) Injury Severity Score (ISS) ≥ 16; (2) current infectious disease; (3) history of brain disorders (e.g., stroke, TBI, brain tumor, or Parkinson’s disease); (4) history of drug abuse; and (5) current pregnancy. The ISS is a medical score used to assess trauma severity based on the worst injury of the six body systems. Each injury in every body region is assigned to an abbreviated injury scale (AIS) on a six-point ordinal scale (1: minor, 6: maximal). To calculate the ISS (ranging from 3 to 75), we selected the highest AIS in each of the three most severely injured body regions, squared each AIS, and added the three squared numbers^58^.

### Study procedure

When a patient with head trauma was identified in the ED or outpatient clinic, physicians of the research team evaluated patient eligibility for the study. Enrolled patients were carefully interviewed, and their medical records were reviewed. Data including demographics (age, sex, height, and weight), injury-related information (the time of injury, mechanism, initial Glasgow Coma Scale scores within 72 h, loss of consciousness [LOC], and post-traumatic amnesia), brain computed tomography findings, medical history, and history of substance abuse were collected from medical records and interviews with the patient or patient’s family.

After enrollment, the patients visited the clinic at multiple time points after injury (< 72 h, at 1 week, 1 month, 3 months, and 6 months). Blood samples were collected within 72 h, at 1 week, 1 month, and 3 months after injury. Since we planned to recruit patients within 1 month after head trauma, blood samples within 72 h or at 1 week could not be obtained depending on the time of enrollment. Blood samples were collected into two 10-mL ethylenediamine tetraacetic acid tubes and three serum separation transport tubes of 15 mL for the serum, centrifuged, aliquoted, labeled, and stored in a freezer at -80 °C until they were transported for analysis at each visit.

Clinical assessments were evaluated at 1 week, 1 month, 3 months, and 6 months after injury. If the patient was enrolled 1 week after injury, clinical assessments at 1 week could not be evaluated.

### Clinical assessments

We evaluated neuropsychological symptoms, including depressive symptoms and cognitive dysfunction. Depressive symptoms were assessed using the BDI-II at 1, 3, and 6 months after injury. The BDI-II is a widely used 21-item self-report inventory that evaluates the severity of depression. Each item is rated on a 4-point scale ranging from 0 to 3. The maximum total score is 63^59^. Cognitive function was assessed using the K-MoCA and FAB at 1 and 3 months. To improve the accuracy of assessing neuropsychological symptoms, several cognitive measures have been created and introduced. Nevertheless, as a result of early post-concussion symptoms, including intense fatigue, sleep disorders, and anxiety, experienced by many patients, we have decided to use the K-MoCA and FAB tests. These tests are less time-consuming, preventing excessively lengthy assessment times. The K-MoCA is a 30-point test that explores the following: short-term memory, visuospatial ability, executive function, attention, concentration, working memory, language, and orientation^60, 61^. The MoCA has been shown to have higher sensitivity for memory testing than the MMSE in mild cognitive impairment^62^. Although MoCA has not had its validity confirmed as a screening tool for cognitive impairment in mTBI, numerous studies of mTBI have used the MoCA for measuring cognitive function^63–66^. The FAB consists of six subtests evaluating conceptualization, mental flexibility, motor programming, sensitivity to interference, inhibitory control, and environmental autonomy. The total FAB score ranges from 0 to 18 and takes approximately 10 min^67^. The FAB was designed to assess frontal lobe function including executive functioning. Patients who experience an mTBI show significantly worse cognitive performance on general cognitive ability, naming, memory, and executive functioning after the injury^68^. Given that the FAB better assesses executive function and has also been employed in previous studies^68^, we selected it as a complementary tool to K-MoCA.

Post-concussive symptoms were evaluated using the RPCQ at 1 week, 1 month, 3 months, and 6 months after injury, where the patient is asked to weigh the severity of 16 post-concussive symptoms compared with premorbid levels, using values from 0 to 4. The total RPCQ score ranges from 0 to 64^69^.

Overall function was evaluated using the GOSE at 1 month, 3 months, and 6 months after injury. The GOSE consists of eight stages: 1, dead; 2, vegetative; 3, lower severe disability; 4, upper severe disability; 5, lower moderate disability; 6, upper moderate disability; 7, lower good recovery; and 8, upper good recovery. The GOSE score was assessed using questions from structured GOSE interviews^70^.

### Outcome

In this study, the main outcome measures were the RPCQ and GOSE at 6 months after injury. A lower RPCQ score and a higher GOSE score suggest better outcomes. When developing a predictive model with a binary outcome variable, RPCQ and GOSE scores were dichotomized into poor and good outcomes. For dichotomization of the RPCQ score, we used the International Classification of Diseases, Tenth Edition (ICD-10) criteria for PCS. The ICD-10 criteria for PCS were used because the ICD-11 criteria had not been released yet when we planned and started the study in January 2018.

Patients with a history of head trauma, usually sufficiently severe to result in LOC, were diagnosed with PCS if they met at least three of the following criteria: (1) headache, (2) dizziness, (3) fatigue, (4) irritability, (5) memory impairment, (6) difficulty concentrating and performing mental tasks, (7) insomnia, and (8) reduced tolerance to alcohol, stress, or emotional excitement^57^. The RPCQ cannot be used to diagnose PCS. In this study, we matched 16 post-concussive symptoms of the RPCQ to 8 symptoms of the ICD-10 criteria (see details in Supplementary Methods). If the value for each symptom was 2 (a mild problem) or more, the patient was considered to have the matched sign of the ICD-10 criteria. Therefore, if the patients’ RPCQ score met the requirements for PCS based on the ICD-10 criteria, they were considered to have a poor RPCQ score. Regarding GOSE, a score < 8 was regarded as a poor GOSE score in this study. Patients with poor RPCQ and GOSE scores at 6 months after injury were classified as having PR and other patients were classified as having GR.

### Proteomic analysis

In total, 117 plasma samples were prepared in block-randomized batches with respect to age, sex, and prognostic recovery group (PR vs. GR) using the “psych” package (version 1.9.12) in R (version 4.0.5; R Foundation for Statistical Computing, Vienna, Austria). The six highly abundant plasma proteins were depleted using a Multiple Affinity Removal System Human-6 column (MARS Hu-6, 4.6 mm × 100 mm; Agilent, Santa Clara, CA, USA). Proteins (100 μg) were digested to tryptic peptides with RapiGest buffer, and digested peptides were desalted using Oasis® HLB cartridges (Waters Corp., Milford, MA, USA). A volume of a 10-µL crude stable isotope-labeled standard (SIS) peptide mixture (JPT Peptide Technology, Acton, MA, USA) was spiked into each 90-µL peptide sample for MRM-MS analysis, a highly selective and reproducible proteomics technique^71^ (see details in Supplementary Methods).

The plasma samples were analyzed using an Agilent 6490 triple quadrupole mass spectrometer (Agilent) with a Jetstream electrospray source coupled to a 1260 Infinity HPLC system (Agilent) (see details in Supplementary Methods). Protein levels were measured using MRM-MS, and raw MRM-MS data were processed using Skyline (MacCoss Lab; University of Washington, Seattle, WA) to compute the peak area of the transitions (see details of analytical target selection in Supplementary Methods)^72^. Each sample was analyzed in single-replicate and block-randomized batches. Targets with interference signals were excluded from the automated detection of an inaccurate and imprecise transitions algorithm (see details in Supplementary Methods)^73^. The quantity of spiked SIS peptides for individual analyses was matched with each endogenous target level in the pooled matrix sample. The peak area ratios (peak areas of endogenous peptides normalized to those of their corresponding SIS peptides) were used to compare the relative abundance of candidate peptides between the samples. The log_10_-transformed peak area ratios were used for data processing. Proteins with high skewness (> 1.5 or < − 1.5) were excluded from data processing.

### Statistical analysis

The skewness of the log-transformed protein levels was assessed using the “e1071” package (version 1.7-3) in R (version 4.0.5). Student’s t-test, χ^2^ test, Pearson’s correlation analysis, and cubic splines function were performed for the longitudinal expression of proteins using base packages in R. Linear regression and logistic regression analyses were performed using the “caret” packages (version 6.0-86) in R. Serial mediation and moderation analyses were performed using the “processR” package (version 0.2.6) and “lavaan” package (version 0.6-9) in R (see details in Supplementary Methods). Gene ontology functional annotation of proteins was performed using DAVID Bioinformatics Resources (version 6.8; https://david.ncifcrf.gov/).

### Supplementary Information

Supplementary Information 1.

Supplementary Tables.

## Data Availability

The raw MRM-MS files for all 117 longitudinal plasma samples from 42 patients were deposited in PeptideAtlas (http://www.peptideatlas.org), along with the quantitation target lists (dataset identifier: PASS01720; password: YN5572u). Data supporting this research are available upon request. Correspondence and material requests should be addressed to Byung-Mo Oh.
