# Supplementary material for: Proteomic discovery of prognostic protein biomarkers for persisting problems after mild traumatic brain injury
Source: Sci Rep. 2023 Nov 13;13:19786. doi: 10.1038/s41598-023-45965-9 (PMC10643618; doi:10.1038/s41598-023-45965-9)

**Title : Proteomic Discovery of Prognostic Protein Biomarkers for Persisting Problems After Mild Traumatic Brain Injury**

Authors: Min-Yong Lee, MD, Minsoo Son, PhD, Hyun Haeng Lee, MD, Min-Gu Kang, MD, Seo Jung Yun, MS, Han Gil Seo, MD, PhD, Youngsoo Kim, PhD, and Byung-Mo Oh, MD, PhD

**Supplementary Information**

**Supplementary Results**

*Abnormal computed tomography findings*

83-year-old-female: small amount of focal subarachnoid hemorrhage at Rt. sylvian cistern lower aspect, and left frontal area

28-year-old male: small amount of acute extraaxial hemorrhage at right frontotemporal convexity

72-year-old female: small acute extraaxial hemorrhage including subarachnoid hemorrhage, subdural hemorrhage and hemorrhagic contusion in left frontal lobe

63-year-old male: possible hemorrhagic contusion by counter-coup injury in the left frontal lobe anterior cranial fossa floor

46-year-old male: small amount of acute extradural hemorrhage or subdural hemorrhage involving bilateral frontal convexity and small amount of acute subarachnoid hemorrhage along right frontal sulci and interhemispheric fissure

58-year-old male: small amount of acute subdural hemorrhage

63-year-old male: small amount of acute extraaxial hemorrhage

**Supplementary Methods**

*Outcome*

We matched 16 post-concussive symptoms of the Rivermead Post-Concussion Symptoms Questionnaire (RPCQ) to 8 symptoms of the International Classification of Diseases, Tenth Edition (ICD-10) criteria for post-concussion syndrome (PCS).

| **Symptoms of ICD-10 criteria for PCS** | **Symptoms of the RPCQ** |
| --- | --- |
| Headache | Headaches |
| Dizziness | Feeling of dizziness, nausea and/or vomiting, double vision, blurred vision |
| Fatigue | Fatigue |
| Irritability | Being irritable |
| Memory impairment | Forgetfulness |
| Difficulty concentrating and performing mental tasks | Poor concentration, taking longer to think |
| Insomnia | Sleep disturbance |
| Reduced tolerance to alcohol, stress, or emotional excitement | Feeling depressed or tearful, feeling frustrated or impatient, noise sensitivity, light sensitivity, restlessness |

*Materials*

Rapigest^TM^ SF was purchased from Waters (Milford, MA, USA). Neat formic acid (FA), dithiothreitol, iodoacetamide, ammonium bicarbonate (ABC), and bicinchoninic acid (BCA) solution were obtained from Sigma-Aldrich (St. Louis, MO, USA). Sequencing-grade modified trypsin was acquired from Promega (Madison, WI, USA). High-performance liquid chromatography (HPLC)-grade water, 0.1% FA in HPLC water, 0.1% FA in acetonitrile, and methanol were purchased from Thermo Fisher Scientific (Bremen, Germany).

*Depletion of high-abundance proteins*

Six high-abundance proteins (albumin, immunoglobulin G, immunoglobulin A, haptoglobin, transferrin, and alpha-1-antitrypsin) were depleted on an HPLC instrument that was coupled to a Multiple Affinity Removal System Human-6 (MARS Hu-6, 4.6 mm × 100 mm, Agilent, CA, USA). A total of 44 μL of each plasma sample was diluted with 176 μL MARS buffer A (Agilent, CA, USA) and filtered using 0.22 μm Spin-X filters (Corning Costar, NY, USA). Then, 200 μL of each diluted sample was injected into the HPLC instrument. The total run time for each run was 28 min. The MARS column was equilibrated with buffer A, and 200 μL of each diluted sample was loaded onto the MARS column at a flow rate of 0.5 mL/min for 10 min. Flow-through fractions, eluted at 3 min and containing low-abundance proteins, were used in subsequent experiments. The bound proteins were eluted for 16.5 min at a flow rate of 1.0 mL/min using 100% buffer B (Agilent, CA, USA). Depleted plasma samples were then concentrated at 3470 *g* for 6 hours at 4 °C using 3000-Da molecular weight cutoff centrifugal filter units (Amicon Ultra-4 3K, Millipore, MA, USA).

*Tryptic digestion and desalting*

The depleted and concentrated protein in the plasma samples was measured using the Pierce^TM^ BCA Protein Assay Kit (Thermo Scientific, Rockford, IL, USA). Then, 20 μL of solution that contained 0.2% RapiGest^TM^, 20 mM dithiothreitol (Merck, Darmstadt, Germany), and 100 mM ABC (pH 8.0) was added to 100 μg of protein and incubated at 60 °C for 60 min with shaking for denaturation and reduction. Next, 10 μL 100 mM iodoacetamide (Sigma, MO, USA) was added and incubated at room temperature for 30 min with shaking in the dark to alkylate the proteins. Subsequently, 40 μL 0.1 μg/μL trypsin (sequencing-grade, Promega, WI, USA) in 50 mM ABC solution (pH 8.0) was added and incubated at 37 °C for 4 hours with shaking to digest the proteins. The digestion was quenched with 10 μL 10% FA at 37 °C for 30 min with shaking.

Quenched samples were centrifuged at 16,602 *g* and 4 °C for 60 min, and 90 μL of the supernatant was transferred to a clean tube to remove any byproducts of the RapiGest. Digested samples were cleaned using Oasis^®^ HLB (hydrophilic-lipophilic balance) 1 cc (30 mg) extraction cartridges (Waters Corp., MA, USA). Columns were activated with 1 mL 100% methanol and equilibrated with 3 mL 100% acetonitrile and 5 mL 0.1% FA in distilled water. Each digested plasma sample was loaded onto the column twice and washed with 3 mL 0.1% FA in distilled water. Samples were eluted with 0.5 mL 0.1% FA in 40% acetonitrile and 0.5 mL 0.1% FA in 60% acetonitrile. Eluates were lyophilized on a vacuum centrifuge and resolubilized to 0.25 μg/μL in 0.1% formic acid/water.

*Liquid chromatography-tandem mass spectrometry*

The plasma samples were analyzed on an Agilent 6490 triple quadrupole (QQQ) mass spectrometer (Agilent, CA, USA) with a Jetstream electrospray source coupled to a 1260 Infinity HPLC system (Agilent, CA, USA). Buffers that consisted of 0.1% formic acid/water (v/v) and 0.1% formic acid/acetonitrile (v/v) were used as mobile phases A and B during the mass analysis. A reversed-phase analytical column (150 mm × 0.5 mm id, Agilent Zorbax SB-C18, 3.5-μm particle size) was used with mobile phases A and B to separate the peptides. In every analysis between batches, liquid chromatography (LC) quality control and a suitability test were performed using the 6 × 5 LC-MS/MS Peptide Reference Mix from Promega (Madison, WI, USA).

The multiple reaction monitoring-mass spectrometry (MRM-MS) analyses were performed in positive mode. A total of 10 μL of the sample was injected into the mass spectrometer for each analysis. The total LC run time was 70 min, and the specific LC buffer system was as follows: The flow rate of the mobile phases was 40 μL/min. The LC gradient started at 97% mobile phase A and 3% mobile phase B. The gradient of mobile phase B was increased linearly from 3% to 40% and flowed for 52 min for the separation of elution of the peptides. The buffer with 60% mobile phase B flowed for 3 min, and the column was equilibrated for the next run for 15 min with the buffer with 3% mobile phase B. The ion spray capillary voltage was 2500 V, and the nozzle voltage was set to 2000 V. The cell accelerator voltage was 5 V, the delta electron multiplier voltage was adjusted to 200 V, and the fragment voltage was set to 380 V. The temperature of the drying gas was set to 250 °C at 15 L/min, and the sheath gas was adjusted to 350 °C at a flow rate of 12 L/min.

*Analytical target selection strategy*

A total of 3130 candidates of target proteins were compiled from three comprehensive traumatic brain injury (TBI) animal model studies that used RNAseq techniques plus original TBI biomarkers as follows: (i) 1828 differentially expressed genes (DEGs) in each brain cell type in the acute phase (24 h) of TBI (Arneson D, et al. *Nat Commun.* 2018;9(1):3894); (ii) 458 DEGs from the enriched brain endothelium of a TBI mouse model in the acute (3 h), subacute (48 h), and chronic (1 month) phases (Munji RN, et al. *Nat Neurosci*. 2019; 22(11):1892-1902); (iii) 1291 DEGs in the brain of a TBI rat model in the chronic phase (3 months) (Lipponen A et al. *Sci Rep*. 2016;6:31570); and (iv) 16 well-known TBI biomarkers (Kim HJ, et al. *JCI Insight.* 2018;3(1):e97105). To convert mouse or rat gene names to human gene names, we used the “biomaRt” package (2.46.3) in R (Version 4.0.5), and only reviewed proteins (UniProtKB/Swiss-Prot) were listed as the candidates.

A FASTA file that contained the sequences of all candidate target proteins was inputted into Skyline (Ver20.1, MacCoss Lab, University of Washington, USA) (MacLean, B. et al. *Bioinformatics* 2010;26(7):966-968), and in silico digestion, with the transition setting, was performed per the following criteria: trypsin proteolytic peptide, with no missed cleavage; peptide length, 6−30 amino acids; exclusion of peptides that contained methionine or RP/KP; structural modification, carbamidomethylation at cysteine; 2+, 3+ charge-state for precursor ions; 1+, 2+ charge-state for y, b fragment ions; mass spectrometry (MS) spectrum range, 10−1400 m/z; and method match tolerance, 0.055 m/z. The transition patterns for each peptide were compared with the National Institute of Standards and Technology reference library (http://peptide.nist.gov/), PeptideAtlas spectral library (http://www.peptideatlas
.org/speclib/), and SWATHatlas spectral library (https://db.systemsbiology.net/sbeams/cgi/PeptideAtlas/
GetDIALibs). We selected 763 peptides representing 528 proteins as detectable targets in plasma digests, with dot-product scores above 0.8, peak intensity greater than 800, and unique tryptic peptides. Crude stable isotope-labeled standard (SIS) peptides (JPT Peptide Technology, Acton, MA, USA) were synthesized to quantify each protein. SIS peptides were synthesized with lysine or arginine that were heavy isotope-labeled (^13^C_6_^15^N_2_ or ^13^C_6_^15^N_4_) at the C-termini. A 537-interference-free peptide set was selected, and the top one transition and peptide per protein was selected for individual sample MRM-MS analysis. Out of 528 proteins, 420 were used for data-processing, according to their skewness (≤ 1.5 and ≥ ‒ 1.5).

*Interference-free check in MRM-MS analyses*

The automated detection of inaccurate and imprecise transitions (AuDIT) algorithm (Abbatiello SE et al., *Clin Chem*. 2010 Feb 1;56(2):291-305) was used to identify contributions from falsely transmitted ions. Transitions that met the criteria of consistent peak areas, repeated analysis [coefficient of variance (CV) < 30%], and an adjusted *P* value >1×10^-5^ in the comparison between the product ion intensity of endogenous peptides and the SIS peptides in the triplicate analyses were selected for further analysis. The pooled plasma digests of the training set samples were used as a matrix. All SIS peptides were spiked at 100-femtomole quantities for the AuDIT analysis.

*Serial mediation and moderation analysis*

The analyses were performed using the “processR” package (Version 0.2.6) and “lavaan” package (Version 0.6-9) in R (Version 4.0.5), according to the guidelines recommended by Andrew F. Hayes (Hayes, A.F., New York: Guilford Press; 2013). Bootstrapped mediation analyses (N=1000) were performed, and the statistical significance of the indirect effect was indicated as the 95% bias-corrected confidence interval (CI) around the estimate of the indirect effect, which did not cross zero. To test the indirect effects between protein levels, neuropsychological assessments, and prognostic outcomes in a simple and clear way, we established the rules for the serial mediation and moderation analyses as follows: (i) single neuropsychological assessment should be used at each time point to test each indirect effect; (ii) an event only could be affected by another event that occurred at earlier time point; (iii) test the effect of protein level on neuropsychological assessments or prognostic outcomes, not the opposite case (i.e. neuropsychological assessments to protein level), to find protein biomarkers that affect the event; and (iv) not consider indirect effects between protein levels.

- The Hayes model #4 was used to test the indirect effect between neuropsychological assessments and prognostic outcomes and that of the protein level at 1 week (or 3 months) on prognostic outcomes, mediating the neuropsychological assessment at 3 months (Figures 1 and 3). The equation of model #4 is as follows:


$$M \sim a*X$$

$$Y \sim b*M + c*X$$

$$Indirect :=a*b; Direct := c$$

$$Total :=direct+indirect$$

- The Hayes model #7 was used to test the moderation of the protein level at 1 month to the indirect effect of neuropsychological assessments at 1 month on the prognostic outcome, mediating neuropsychological assessments at 3 months (Figure 3). The equation of model #7 is as follows:


$$M \sim a1*X + a2*W + a3*X:W$$

$$Y \sim c*X + b*M$$

$$W \sim W.mean*1$$

$$W \sim\sim W.var*W$$

$$Indirect := a1 + a3*W.mean*b$$

$$Direct :=c$$

$$Total := Direct + Indirect$$

$$Index.mod.med := a3*b$$

- The Hayes model #14 was used to test the moderation of the protein level at 3 months to the indirect effect of neuropsychological assessments at 1 month on the prognostic outcome, mediating neuropsychological assessments at 3 months (Figure 3). The equation of model #14 is as follows:


$$M \sim a*X$$

$$Y \sim c*X + b1*M+b2*W+b3*M:W$$

$$W \sim W.mean*1$$

$$W \sim\sim W.var*W$$

$$Indirect := a*b1 + b3*W.mean$$

$$Direct :=c$$

$$Total := Direct + Indirect$$

$$Index.mod.med := a*b3$$

*Association of the acute phase events with the biological terms in chronic phases*

To verify the effect of acute events to the biological terms enriched in chronic phases, from 1 week to 3 months, the functional annotation analysis was performed using the proteins which were significant in the serial mediation and moderation analysis and correlated with the acute protein factor (Pearson’s |*r*| < 0.1) (Supplementary Fig. 2c-e and Supplementary Table 6). In the early chronic phase, 14 out of 27 proteins (51.9%), which indirectly affected RPCQ, were significantly associated with the terms affecting prognosis through the “K-MoCA” path, such as neurofilament bundle assembly (EASE score, *P*=0.0023) and axon (EASE score, *P*=0.011) and the terms linked to prognosis through the “BDI” path, such as inflammatory response (EASE score, *P*=0.0028), cellular response to (LPS) (EASE score, *P*=0.0033), and innate immune response (EASE score, *P*=0.0039). In 12 out 18 proteins (66.7%) for Glasgow Outcome Scale-Extended (GOSE), the inflammatory terms, such as growth factor activity (EASE score, *P*=1.35e-4), positive regulation of macrophage chemotaxis (EASE score, *P*=0.0072), and inflammatory response (EASE score, *P*=0.024) and the apoptosis term mitochondrion (EASE score, *P*=0.041) were significantly enriched. In addition to the early chronic phase, the proteins in chronic phases 1 and 2, which indirectly affected the prognostic outcome, were correlated with the acute protein factor and still significantly associated with the biological terms for mTBI. In chronic phase 1, 3 out of 8 proteins (37.5%) for RPCQ and 4 out of 8 proteins for GOSE (50.0%) were associated with the terms such as myelin sheath (EASE score, *P*=0.017), cell aging (EASE score, *P*=0.0045), and neuronal cell body (EASE score, *P*=0.050). In chronic phase 2, 8 out of 20 proteins (40.0%), which indirectly affected RPCQ via the “BDI” path, were associated with the terms for depressive disorder, such as hormone (EASE score, *P*=4.36e-4), ghrelin (EASE score, *P*=0.032), glucose homeostasis (EASE score, *P*=0.041), and long-term depression (EASE score, *P*=0.046). Meanwhile, 8 out of 21 proteins (38.1%) influencing GOSE secondhand through the “FAB” path exposed terms such as apolipoprotein binding (EASE score, *P*=0.0058), IFNγ production (EASE score, *P*=0.019), lipoprotein (EASE score, *P*=0.031), and cellular response to LPS (EASE score, *P*=0.046).

**Supplementary Tables**

**Supplementary Table S1. The 3130-candidate target protein list.**

**Supplementary Table S2. The 420-target protein list and information on the MRM-MS analyses.**

**Supplementary Table S3. Linear regression between the clinical measurements and prognostic outcome.**

**Supplementary Table S4. Indirect effects or moderating indirect effects of proteins on the prognostic outcomes.**

**Supplementary Table S5. The biological terms enriched in neuropsychological paths mediating prognostic outcomes in chronic phases.**

**Supplementary Table S6. The biological terms enriched by the chronic phase proteins associated with acute phase events.**

**Supplementary Table S7. Univariate and multivariate regression for the prognostic group prediction.**

**Supplementary Figures**

**Supplementary Fig. 1 The biological terms enriched in neuropsychological paths mediating prognostic outcomes in chronic phases.** The enriched biological terms by the proteins at 1 week (**a, b**), 1 month (**c**), and 3 months (**d, e**), which affect the prognostic outcome via BDI-II, K-MoCA, and FAB paths. The enriched biological terms from the functional annotation chart (EASE score < 0.1) are depicted, and the scale of the EASE score is depicted on the X-axis of the charts. BDI-II, Beck Depression Inventory-II; K-MoCA, Korean Montreal Cognitive Assessment; FAB, Frontal Assessment Battery; RPCQ, Rivermead Post-concussion Symptoms Questionnaire; GOSE, Glasgow Outcome Scale-Extended.


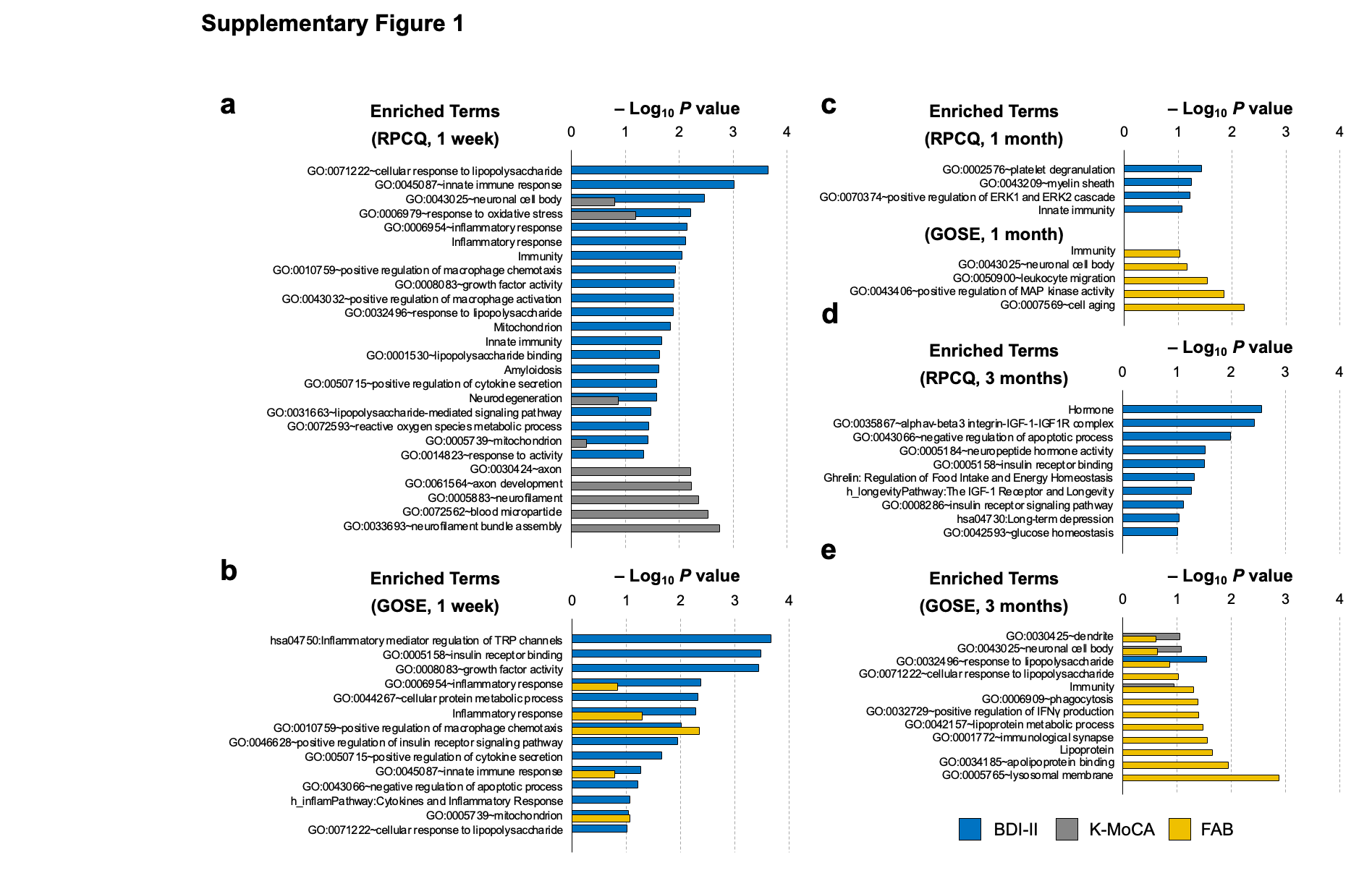


**Supplementary Fig. 2 Characterization of the association between the acute protein factor and the biological events in chronic phases.** (**a)** Functional association network between 19 proteins and GFAP and UCHL1. The network nodes are the proteins, and the size of the nodes denotes the significance of correlation with the acute protein factor. The line between the nodes signifies that the corresponding proteins encoded by the genes could interact physically (STRING database score > 0.3). (**b**) The linear regression for prognostic outcomes using acute protein factor. The red circle represents the poor recovery group, and blue circle represents the good recovery group. Estimates and 95% CIs are marked with R-squared value and significance of regression. Pearson’s correlation between acute protein factor and the significant recovery proteins at 1 week (**c**), 1 month (**d**), and 3 months (**e**). The significant biological terms (EASE score, *P* < 0.05) are depicted on the upper right side of the charts. Bar color denotes the associated neuropsychological assessments. The significance level of Pearson’s correlation is represented as # for *P*<0.1 and * for *P*<0.05. GFAP, glial fibrillary acidic protein; UCH-L1, ubiquitin carboxy-terminal hydrolase L1; BDI-II, Beck Depression Inventory-II; K-MoCA, Korean Montreal Cognitive Assessment; FAB, Frontal Assessment Battery; RPCQ, Rivermead Post-concussion Symptoms Questionnaire; GOSE, Glasgow Outcome Scale-Extended; LPS, lipopolysaccharide; PPI, protein-protein interaction.

**
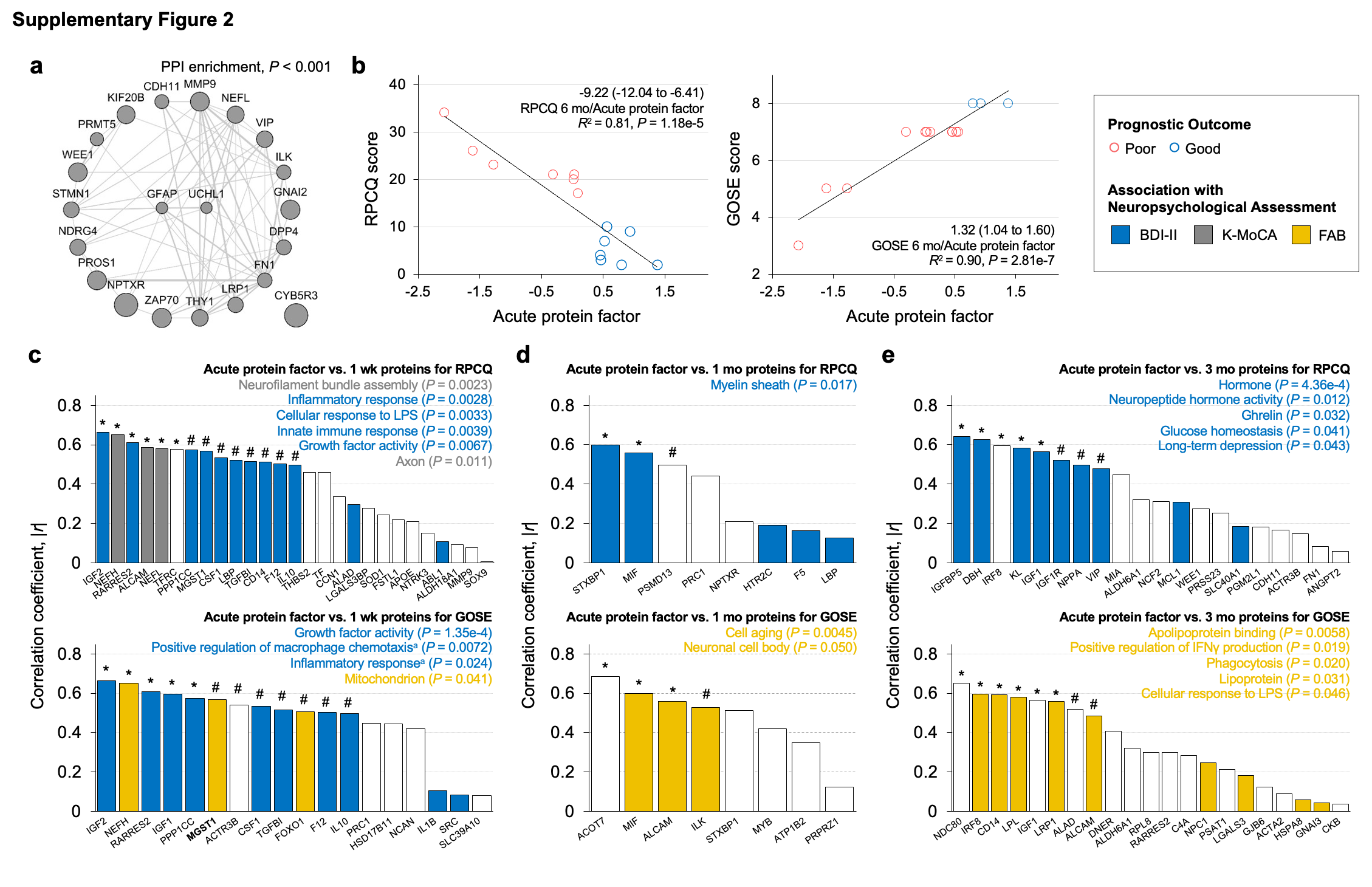
**

**Supplementary Fig. 3 Prognostic performance of protein biomarkers.** The receiver operating characteristic (ROC) curves of cross-validated logistic regression model to predict prognosis of patients with mTBI. The results of multivariate regression models are depicted, except for univariate analysis results of three of each protein in the acute phase. AUC, area under the curve; CI, confidence interval


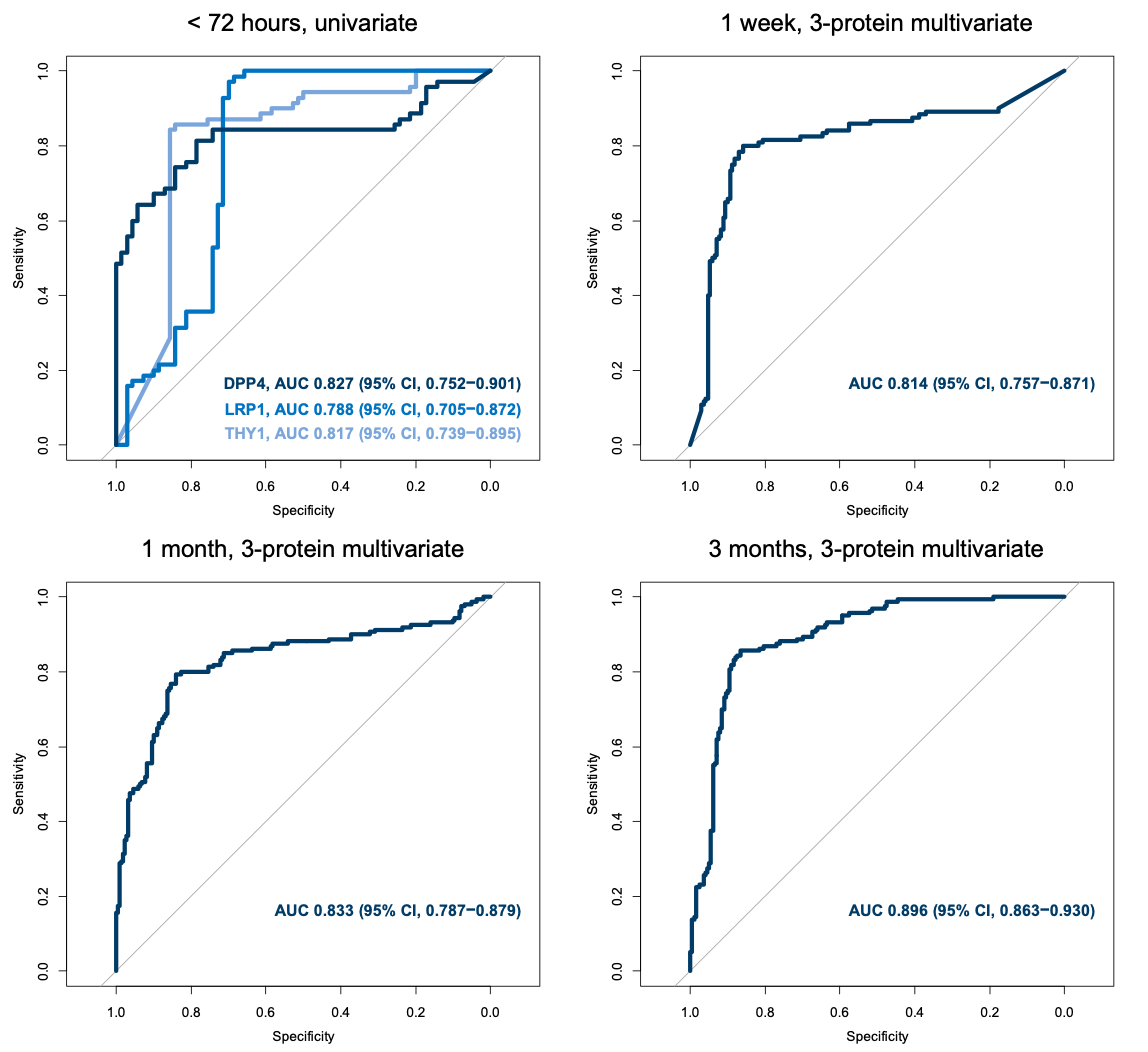


**Supplementary Fig. 4 The longitudinal expression of protein factors in the models.** The longitudinal alterations of the proteins selected in the logistic regression during a follow-up period. The charts for the two prognostic groups are represented. Each dot denotes the mean protein level, and each error bar denotes the 95% confidence interval. Lines between the dots are depicted using splines.


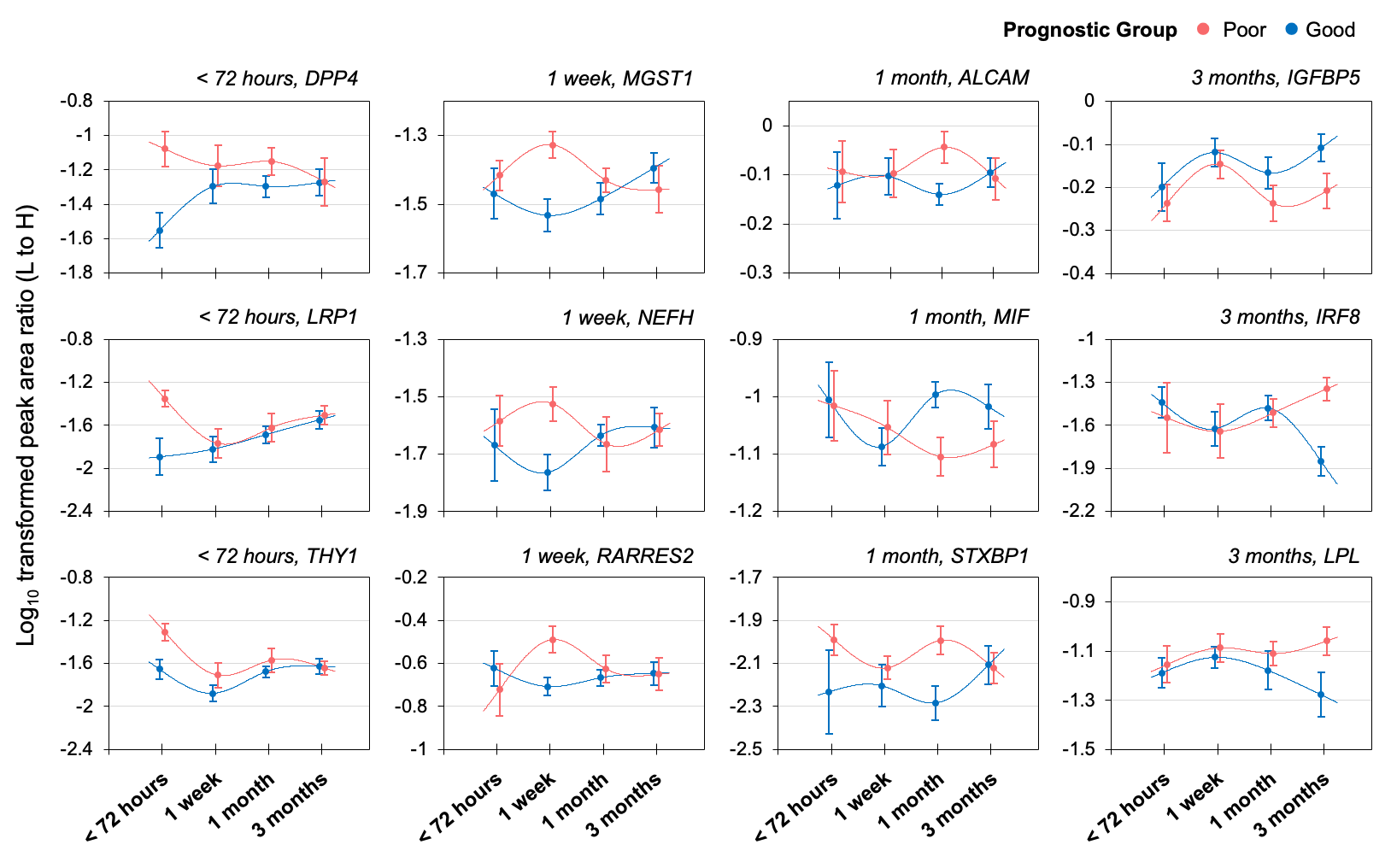

Supplement: Supplementary file 1 — Supplementary Information 1. [file 41598_2023_45965_MOESM1_ESM.docx]
